# Microarray analysis reveals the inhibition of intestinal expression of nutrient transporters in piglets infected with porcine epidemic diarrhea virus

**DOI:** 10.1038/s41598-019-56391-1

**Published:** 2019-12-24

**Authors:** Junmei Zhang, Di Zhao, Dan Yi, Mengjun Wu, Hongbo Chen, Tao Wu, Jia Zhou, Peng Li, Yongqing Hou, Guoyao Wu

**Affiliations:** 1 Hubei Key Laboratory of Animal Nutrition and Feed Science, Wuhan Polytechnic University, Wuhan, 430023 China; 2 Department of Animal Science, Texas A&M University, College Station, Texas 77843 USA

**Keywords:** Biochemistry, Molecular biology

## Abstract

Porcine epidemic diarrhea virus (PEDV) infection can induce intestinal dysfunction, resulting in severe diarrhea and even death, but the mode of action underlying these viral effects remains unclear. This study determined the effects of PEDV infection on intestinal absorption and the expression of genes for nutrient transporters via biochemical tests and microarray analysis. Sixteen 7-day-old healthy piglets fed a milk replacer were randomly allocated to one of two groups. After 5-day adaption, piglets (n = 8/group) were orally administrated with either sterile saline or PEDV (the strain from Yunnan province) at 10^4.5^ TCID_50_ (50% tissue culture infectious dose) per pig. All pigs were orally infused D-xylose (0.1 g/kg BW) on day 5 post PEDV or saline administration. One hour later, jugular vein blood samples as well as intestinal samples were collected for further analysis. In comparison with the control group, PEDV infection increased diarrhea incidence, blood diamine oxidase activity, and iFABP level, while reducing growth and plasma D-xylose concentration in piglets. Moreover, PEDV infection altered plasma and jejunal amino acid profiles, and decreased the expression of aquaporins and amino acid transporters (L-type amino acid transporter 1, sodium-independent amino acid transporter, B(°^,+^)-type amino acid transport protein, sodium-dependent neutral amino acid transporter 1, sodium-dependent glutamate/aspartate transporter 3, and peptide transporter (1), lipid transport and metabolism-related genes (lipoprotein lipase, apolipoprotein A1, apolipoprotein A4, apolipoprotein C2, solute carrier family 27 member 2, solute carrier family 27 member 4, fatty acid synthase, and long-chain acyl-CoA synthetase (3), and glucose transport genes (glucose transporter-2 and insulin receptor) in the jejunum. However, PEDV administration increased mRNA levels for phosphoenolpyruvate carboxykinase 1, argininosuccinate synthase 1, sodium/glucose co-transporter-1, and cystic fibrosis transmembrane conductance regulator in the jejunum. Collectively, these comprehensive results indicate that PEDV infection induces intestinal injury and inhibits the expression of genes encoding for nutrient transporters.

## Introduction

Porcine epidemic diarrhea (PED) virus (PEDV), known as the pathogen of PED, has catastrophic impacts on the global pig industry and causes great economic losses to the world^1^. PEDV, which is an enveloped, single-stranded, positive-sense RNA virus^1,2^, can spread mainly through the fecal-oral route as well as airborne transmission under specific conditions^3^. Since PED was first determined in England in the 1970s, it had spread rapidly from Europe to America, Canada and Mexico, and then recurred in Asian countries, such as Japan and South Korea^2,4,5^. PEDV infection can occur in all ages of pigs, but neonatal piglets are most susceptible and sensitive to the virus, characterized by acute watery diarrhea, vomiting, and accompanied by anorexia and depression, dehydration, and even death^6–9^. In May 2013, PEDV outbreaks in America caused more than the death of 8 million newborn piglets during an epidemic year^10–12^. From the late 2010s, an increasing number of pig farms suffered from the PED-induced huge losses^13^. Given the severity of PED and the lack of effective protective measures, extensive and in-depth studies of PED should be carried out to better understand its pathology.

The gastrointestinal tract is the portal for the digestion and absorption of nutrients, and also plays a vital role in the body’s immune function. Barrier integrity of the intestinal epithelium can protect the intestine from foreign materials and allow nutrients to cross the epithelium^14^. Post-weaning piglets are susceptible to environmental, physiological and even nutritional stresses. Due to its incomplete development of the digestive and immune systems, they are more vulnerable to pathogenic bacteria and viruses, leading to compromised intestinal function and severe diarrhea. PEDV strains are highly enteropathogenic and can impair pig intestinal barrier integrity and crypt stem cell proliferation^15^, reduce the expression of tight junction proteins^16,17^, and accordingly inhibit the growth performance of pigs. Previous studies identified the molecular characterization of PEDV^18,19^ and host pathological responses to the virus^20,21^. However, the mechanisms by which PEDV induces the alteration of specific functions of the gastrointestinal tract, such as nutrient absorption, are largely unknown in nursery pigs. We hypothesized that intestinal dysfunction caused by PEDV infection is related to impaired nutrient transport. To test this hypothesis, the present study was conducted to investigate gene expression profiles of nutrient transporters in the small intestine of pigs infected with PEDV. Our findings are expected to provide a theoretical basis for PED prevention and treatment.

## Results

### Average daily weight gain and diarrhea incidence in piglets

Data on the body weight of piglets are shown in Table 1. Compared with pigs in the control group, PEDV infection decreased final body weight (*P* < 0.05), but increased (*P* < 0.001) diarrhea incidence in piglets. Of note, no piglets in the control group had diarrhea but about 50% of piglets in the PEDV group had this intestinal disorder.

**Table 1:** Effects of PEDV infection on the body weight and diarrhea incidence in piglets. Data are mean ± SD, n = 8.

Items	Control group	PEDV group	*P* value
Initial body weight (kg)	3.45 ± 0.42	3.48 ± 0.44	0.873
Final body weight (kg)	3.89 ± 0.19^a^	3.52 ± 0.50^b^	0.033
Diarrhea incidence (%)	0^a^	54.2^b^	<0.001

^a,b^Means within rows with different superscripts differ (*P* < 0.05).

### Biochemical indices

Data on biochemical indices are shown in Table 2. In comparison with the control group, PEDV infection increased the concentrations of blood urea nitrogen (BUN), chloride and intestinal fatty acid-binding protein (iFABP), and diamine oxidase (DAO) activity (*P* < 0.05), but decreased (*P* < 0.05) the concentrations of high-density lipoprotein (HDL) and D-xylose, in the plasma of piglets. In contrast, PEDV infection did not affect the concentrations of total cholesterol, glucose and total calcium in plasma, compared with the control group.

**Table 2:** Effects of PEDV infection on plasma biochemical indices in piglets. *BUN* blood urea nitrogen, *HDL* high density lipoprotein, *LDL* low density lipoprotein, *DAO* diamine oxidase, *i-FABP* intestinal fatty acid binding protein. Data are mean ± SD, n = 8.

Items	Control group	PEDV group	*P* value
BUN (mmol/L)	0.90 ± 0.09^b^	4.08 ± 0.58^a^	<0.001
Total calcium (mmol/L)	10.1 ± 0.15	9.77 ± 0.57	0.217
Total chloride (mmol/L)	99.9 ± 2.03^b^	104 ± 1.99^a^	<0.001
Glucose (mmol/L)	5.83 ± 0.95	6.59 ± 1.4	0.222
HDL (mmol/L)	3.02 ± 0.45^a^	1.67 ± 0.29^b^	<0.001
LDL (mmol/L)	1.11 ± 0.21	0.94 ± 0.22	0.149
Total cholesterol (mmol/L)	2.25 ± 0.12	2.43 ± 0.52	0.362
DAO (U/L)	8.74 ± 1.68^b^	17.4 ± 2.81^a^	<0.001
D-xylose (mmol/L)	0.73 ± 0.12^a^	0.30 ± 0.06^b^	<0.001
i-FABP (pg/mL)	343 ± 37.4^b^	582 ± 108^a^	<0.001

^a,b^Means within rows with different superscripts differ (*P* < 0.05).

### Concentrations of free amino acids and urea in the plasma of piglets

**Figure 1:**
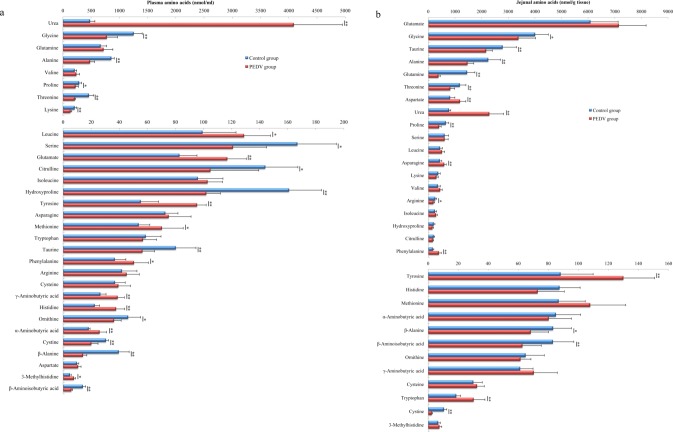
PEDV infection alters the profiles of plasma (**a**) and jejunal mucosa (**b**) amino acids in piglets. Data are mean ± SD, n = 8. *P < 0.05, **P < 0.01.

PEDV infection altered the profiles of amino acids in plasma as illustrated in Fig. 1a. Compared with the control group, PEDV infection decreased (*P* < 0.05) the concentrations of alanine (44%), citrulline (27%), cystine (34%), glycine (38%), hydroxyproline (36%), lysine (36%), ornithine (22%), proline (22%), serine (27%), taurine (30%), threonine (54%), β-alanine (64%), and β-aminoisobutyric acid (57%), while increasing (*P* < 0.05) the concentrations of glutamate (29%), histidine (40%), leucine (23%), methionine (24%), phenylalanine (27%), tyrosine (42%), α-aminobutyric acid (30%), γ-aminobutyric acid (32%), 3-methylhistidine (39%), and urea (752%) in the plasma of piglets.

### Concentrations of free amino acids and urea in the jejunal mucosa of piglets

Effects of PEDV infection on the concentrations of free amino acids in the jejunal mucosa of piglets are summarized in Fig. 1b. In comparison with the control group, PEDV infection elevated (*P* < 0.05) the concentrations of asparagine (38%), aspartate (45%), phenylalanine (133%), tyrosine (48%), tryptophan (62%), and urea (194%), while reducing (*P* < 0.05) the concentrations of alanine (35%), arginine (26%), cystine (77%), glutamine (74%), glycine (16%), proline (38%), taurine (22%), threonine (30%), β-alanine (18%), and β-aminoisobutyric acid (24%). PEDV infection had the tendency (0.1 > *P* > 0.05) to decrease the concentrations of citrulline (14%), hydroxyproline (22%), lysine (18%) and histidine (17%), but to increase the concentrations of glutamate (15%), methionine (20%), and valine (18%), in the jejunal mucosa.

### Gene expression profiles

Porcine Gene 1.1 ST Arrays (Affymetrix, Santa Clara, CA, USA) were used to identify changes in gene expression in response to PEDV infection. We identified 120 annotated transcripts (with complete gene symbol, description, fold change and *P*-value) that were differentially expressed between the control and PEDV groups at the cutoff criteria of the fold change >1.5 and *P* < 0.05. Among these genes, 28 genes were up-regulated and 92 genes were down-regulated (details in Supplementary File S2). IPA (Ingenuity Pathway Analysis) revealed that a large number of genes were associated with nutrient transport and metabolic regulators, involving the transport of water and ions, as well as the absorption, metabolism and transport of other nutrients.

### Biofunctional analysis

Results of the IPA analysis showed that differentially expressed genes were categorized on the basis of functions and related diseases (details in Supplementary File S3). According to functional classification, the significantly altered genes were divided into four distinctive functional categories (see Table 3): (1) molecular transport, (2) amino acid metabolism and related metabolites, (3) infectious diseases, and (4) synthesis of lipids (*P* < 0.05, see Table 3).

**Table 3:** Diseases or functions related to genes differentially expressed in the jejunum of control and PEDV-infected piglets.

Categories	Diseases or Functions Annotation	*P*-value	Predicted Activation State	z-score	Molecules
Molecular transport	Transport of molecules	0.0000000000297	Decreased	−2.841	ABCC2, ABCG2, ABCG5, ACE, ACSL3, ANO6, APOA1, APOB, CD3G, CLCA4, CYBRD1, EXPH5, FLVCR1, MFSD2A, MTTP, MX2, NPC1L1, NRG1, PDZK1, PIGR, PRLR, SLC15A1, SLC1A1, SLC25A15, SLC27A4, SLC39A14, SLC3A1, SLC4A4, SLC5A1, SLC6A19, SLC6A8, SLC9A2, TCN2, TRPA1
Amino acid metabolism, molecular transport, small molecule biochemistry	Transport of amino acids	0.00000104	Decreased	−2.517	ABCC2, ABCG2, SLC1A1, SLC25A15, SLC3A1, SLC6A19, SLC6A8
Infectious diseases	Viral infection	0.000802	Decreased	−2.351	ABCC2, ACE2, ANPEP, APOA1, APOB, AREG, CES1, DMBT1, DPP4, EPHB2, FABP1, GBP1, GCLC, HMGCS1, IFIT3, IFITM3, KRT18, LPL, LRAT, MX1, MX2, NPC1L1, NRG1, PCK1, PGM1, SELL, SPP1, TIMP1
Lipid metabolism, small molecule biochemistry	Synthesis of lipids	0.00496	—	−1.976	ANPEP, APOA1, CERS6, CES1, DPP4, GK, HMGCS1, LPL, MFSD2A, NPC1L1, SPTLC3, SQLE

### Canonical pathways

**Figure 2:**
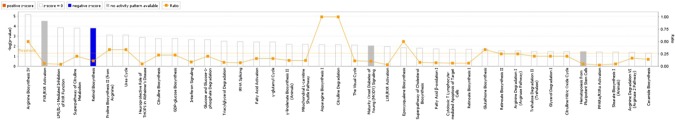
The top 20 signaling pathways in PEDV-infected piglets by the IPA analysis. The higher the histogram, the greater the significance of the pathway. Orange polyline represents the proportion of differentially expressed molecules to the total number of molecules in the pathway.

To determine whether PEDV infection exerted detrimental effects on intestinal function through regulating potential signaling pathways, the IPA core analysis was used to further analyze the microarray data. The differentially expressed genes (DEGs) were categorized into related canonical pathways. The top 20 signaling pathways are shown in Fig. 2. By analyzing the enrichment of differentially expressed genes, we found that several pathways were regulated by PEDV infection, such as citrulline and arginine biosynthesis, interferon signaling, GDP-glucose biosynthesis, fatty acid activation, proline biosynthesis (from arginine), urea cycle, glucose, and glucose-1-phosphate degradation. Among these pathways, PEDV infection greatly affected lipid metabolism and amino acid biosynthesis in piglets.

### Gene networks

**Figure 3:**
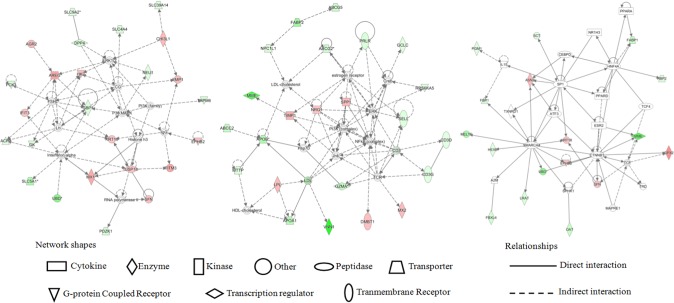
Proposed Top 3 gene networks regulated by PEDV in the jejunum of piglets (built with the IPA tool).

Based on the analysis of differentially expressed genes in the jejunum of PEDV-challenged piglets, gene networks were built with an IPA tool to connect key genes with enriched categories of diseases and functions. Gene networks as well as their related diseases and functions in PEDV-infected piglets are proposed in Supplementary File S4. The three significant networks (Fig. 3) of interest with score >20 corresponded to (1) digestive system development and function, gastrointestinal disease, dermatological diseases and conditions; (2) cardiovascular disease, organ injury and abnormalities, and lipid metabolism; (3) cell death and survival, lipid metabolism, and molecular transport. Notably, nutrient transport as well as lipid and amino acid metabolism may play an important role in the PEDV-induced intestinal dysfunction. Additionally, several genes involved in these networks were further analyzed by qRT-PCR.

### Confirmation of differentially expressed genes by qRT-PCR

**Figure 4:**
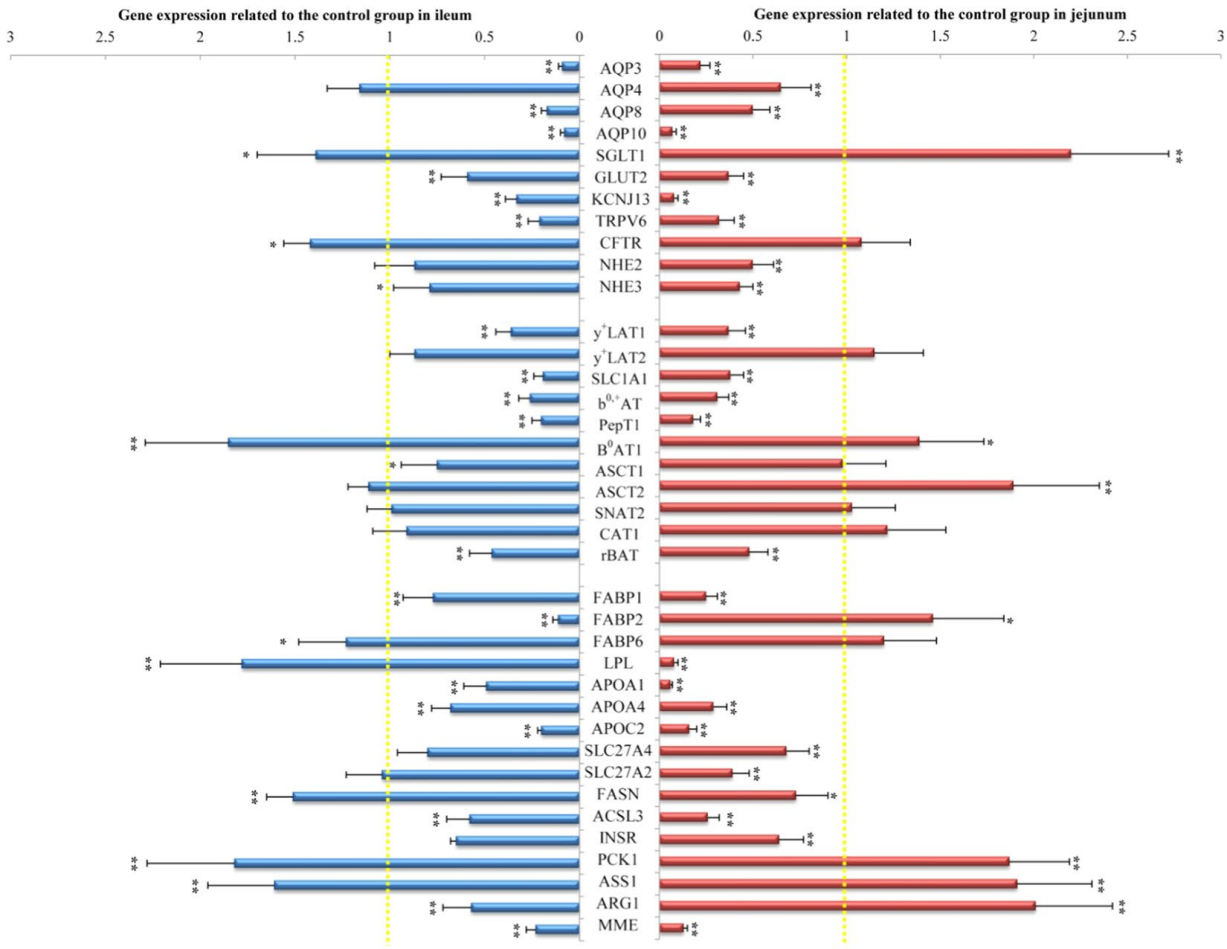
Gene expression in the jejunum and ileum of piglets in control and PEDV-infected groups. All mRNA levels in the control group was normalized to be 1.00. Data are mean ± SD, n = 8. *P < 0.05, **P < 0.01.

To validate the microarray results and confirm the effect of PEDV infection on the expression of genes associated with intestinal nutrient transport and metabolism in piglets, several genes with significant changes were further determined by qRT-PCR in the jejunal and ileal mucosa (Fig. 4). PEDV infection decreased (*P* < 0.05) the mRNA levels for genes associated with water and ion transport, including aquaporin (AQP) 3, AQP4, AQP8, AQP10, potassium inwardly-rectifying channel subfamily J member 13 (KCNJ13), glucose transporter-2 (GLUT2), transient receptor potential cation channel subfamily v Member 6 (TRPV6), sodium/hydrogen exchanger (NHE) 2, and NHE3, while increasing (*P* < 0.05) the mRNA level of sodium/glucose co-transporter-1 (SGLT1). PEDV infection also down-regulated (*P* < 0.05) the expression of genes related to lipid transport and metabolism, including fatty acid binding protein (FABP) 1, lipoprotein lipase (LPL), apolipoprotein (APO) A1, APOA4, APOC2, solute carrier family 27 member 4 (SLC27A4), solute carrier family 27 member 2 (SLC27A2), fatty acid synthase (FASN), and long-chain acyl-CoA synthetase 3(ACSL3), while increasing (*P* < 0.05) FABP2 mRNA level in the jejunum. Moreover, PEDV infection reduced (*P* < 0.05) mRNA levels for L-type amino acid transporter 1 (y^+^LAT1), sodium-dependent glutamate/aspartate transporter 3 (SLC1A1), sodium-independent amino acid transporter (b^0,+^AT), peptide transporter 1 (PepT1), and B(^0,+^)-type amino acid transport protein (rBAT), but elevated (*P* < 0.05) those for system B(0) neutral amino acid transporter 1 (B^0^AT1) and sodium-dependent neutral amino acid transporter 2 (ASCT2). Additionally, PEDV infection increased (*P* < 0.05) mRNA levels for phosphoenolpyruvate carboxykinase 1 (PCK1), argininosuccinate synthase 1 (ASS1) and arginase 1 (ARG1), but decreased (*P* < 0.05) those for insulin receptor (INSR) and membrane metalloendopeptidase (MME) in the jejunal mucosa of piglets.

Similarly, PEDV infection decreased (*P* < 0.05) mRNA levels for ileal AQP3, AQP8, AQP10, KCNJ13, GLUT2, TRPV6, and NHE3, but increased (*P* < 0.05) those for SGLT1 and CFTR in piglets. mRNA levels for genes associated with lipid transport and metabolism, including FABP1, FABP2, APOA1, APOA4, APOC2, and ACSL3 were reduced (*P* < 0.05), whereas those for FABP6, LPL and FASN were increased (*P* < 0.05) in the ileum of PEDV-infected piglets, compared with control piglets. Moreover, PEDV could decrease the expression of genes encoding for amino acid transporters, such as y^+^LAT1, SLC1A1, b^0,+^AT, PepT1, ASCT1, and rBAT. On the contrary, PEDV infection up-regulated (*P* < 0.05) B^0^AT1 expression in the ileum of pigs. In addition, PEDV infection decreased (*P* < 0.05) mRNA levels for INSR, ARG1, and MME, but increased (*P* < 0.05) those for PCK1 and ASS1 in the ileum of piglets.

## Discussion

In the present study, microarray analysis was applied to elucidate the mechanism responsible for intestinal dysfunction induced by PEDV infection. There were 120 differentially expressed genes between the PEDV and control groups. Those genes were classified into three categories: molecular transport, amino acid metabolism, and infectious diseases. The IPA analysis revealed that these differentially expressed genes were primarily involved in pathways including interferon signaling, water and ion transportation, nutrient (glucose, lipids and amino acids) absorption, metabolism and transport. Therefore, the expression of intestinal nutrient transporters and subsequently the absorptive function may be dramatically altered in response to PEDV infection. To our knowledge, this is the first study to quantify intestinal gene expression in PDEV-infected pigs by using microarray analyses.

There is very little information available on the regulation of intestinal transporters in piglets by PEDV infection^22–25^. Schweer^26^ found that PEDV infection decreased the GLUT2 mRNA level but increased the SLC6A14 level in the piglet jejunum. In addition, Wang *et al*.^9^ reported reductions in the mRNA levels for FABP2 in the jejunum of PDEV-infected piglets. Our results are consistent with those from these two studies although SLC6A14 expression was not determined in the present study. Regarding the omic studies with PEDV-infected pigs, Sun *et al*.^22^ found that PEDV infection enhanced the expression of 7044 genes and most of them were related to the immune response to infectious disease pathways. Of note, based on the KEGG analysis, 374 of the differentially expressed genes (DEGs) are related to nutrient transport and catabolism, and this result is in good agreement with that of our present study. Similarly, an *in vitro* study involving the RNA-seq analysis of Vero E6 cells revealed that the DEGs were mainly related to the immune response and the mTOR signaling pathway that play a vital role in PEDV antiviral regulation^23^. Likewise, results of proteomic analysis indicated that PEDV infections in pigs altered the abundance of several jejunal proteins involved in cell function and immune response^24^.

Intestinal integrity plays a critical role in preventing luminal bacteria and maintaining gut barrier function^25,27^. PEDV has been reported to rapidly invade the porcine enterocytes^6^ and cause small intestinal dysfunction, resulting in severe diarrhea, growth retardation, and even death^11^ in piglets. The present study observed that PEDV induced a decrease in average daily gain, but an increase in diarrhea incidence, which was consistent with the previous studies^9,14,28^. Besides, concentrations of D-xylose and i-FABP were decreased, whereas DAO activity was increased in plasma. Because plasma D-xylose, DAO, and i-FABP have been recognized as biomarkers for assessing intestinal barrier function and integrity^9,25,29^, decreased D-xylose along with elevated DAO and i-FABP in plasma indicated that PEDV administration could induce intestinal damage and impair intestinal function in piglets. This notion was corroborated by the reduction in plasma citrulline concentration, which serves as an indicator of intestinal absorption function^6,9,30^.

There were 120 DGEs between the control and PEDV groups, most of them were classified by the IPA analysis into molecular transport, amino acid metabolism, infectious diseases, lipid metabolism, and viral infection. In the light of the canonical pathways and networks built with IPA tools, we attached great importance to the regulation, by PEDV infection, of genes involved in nutrient transport and metabolism.

Firstly, PEDV infection may affect water and ion transport in the small intestine. The expression of genes encoding for water channels, such as AQP3, AQP8, and AQP10 was decreased in both jejunal and ileal mucosae after PEDV infection. Meanwhile, the expression of genes encoding for ion transports was also regulated by PEDV. Specifically, KCNJ13, which encodes for a potassium channel family protein that allows potassium ions to pass into cells^31^, and TRPV6, which functions as a membrane calcium channel^32^, were down-regulated; but CFTR, a chloride channel^33^, was up-regulated in PEDV-infected piglets. Zhang *et al*.^34^ reported that elevation of CFTR expression is usually associated with clinical secretory diarrhea. Recently, PEDV infection has also been reported to remarkably decrease the activities of Na^+^-K^+^-ATPase and Ca^2+^-Mg^2+^-ATPase in enterocytes, thereby inducing abnormal Na^+^, K^+^, Ca^2+^, and Mg^2+^ distribution^35^. Besides, PEDV infection down-regulated the expression of the NHE3 gene, which plays a significant part in NaCl reabsorption^36^. The expression and activity of NHE3 were usually significantly inhibited in inflammatory bowel disease and cholera toxin-induced diarrhea^37,38^. Interestingly, chloride plays an important role in maintaining electrolyte balance and blood osmotic pressure; therefore, the elevation of chloride in plasma may be attributed to an impairment of intestinal chloride transport and the severe dehydration of PEDV-infected piglets. Collectively, PEDV may induce water and ion imbalances, and consequently result in severe diarrhea and dehydration in piglets.

Secondly, PEDV infection affected glucose transport and metabolism in the small intestine. Glucose can be transported into small intestinal cells by SGLTl^39,40^. Then intracellular glucose is transported across the intestinal cell basement membrane into the blood circulation by GLUT2 to maintain the balance of blood glucose level^41^. There are extensive studies regarding the effects of virus on the expression of glucose transporters. Both hepatitis C virus (HCV) and Mayaro virus (MAYV) infection can down-regulate GLUT1/2 gene expression, causing the inhibition of glucose absorption^42,43^. However, the infection of human cytomegalovirus (HCMV) and Kaposi’s sarcoma-associated herpes virus (KSHV) can up-regulate GLUT 1/3/4 expression and increase the capacity of virus-infected cells to transport glucose^44,45^. The discrepancy in the expressions of intestinal glucose transporters may be attributed to the differences in virus types and experimental animals. In the present study, PEDV increased expression of SGLT1 but decreased expression of GLUT2 in the piglet small intestine, indicating that PEDV induced a disorder of glucose transport in the gut. George *et al*.^46^ reported that activated INSR can stimulate the PI3-K signaling pathway, which could not only promote glucose transport by GLUTs, but also inhibit gluconeogenesis by reducing PCK1 that encodes for a key enzyme of gluconeogenesis PEPCK-C^47^. Similarly, the down-regulated expression of the INSR gene, but up-regulated expression of the PCK1 gene were observed in the small intestine after PEDV infection. Therefore, we surmise that PEDV may alter intestinal glucose absorption and metabolism by regulating GLUT, SGLT1, INSR, and PCK1 expression in pigs.

Thirdly, PEDV infection could affect the intestinal amino acid transport and metabolism. Amino acids are preferential nutrients for the intestinal tract to synthesize intestinal proteins, polyamines, nitric oxide, ATP, and other products with enormous biological function^48–50^. In the present study, amino acid profiles in both plasma and jejunum were dramatically altered by PEDV infection. Specifically, basic amino acid (histidine, lysine, and arginine) concentrations were reduced in the jejunal mucosa of PEDV-infected piglets. Arginine is considered to be the nitrogenous precursor for the synthesis of nitric oxide and as a regulator to prevent cell death and promote protein synthesis^51–53^. It can be synthesized from citrulline in plasma and from ornithine in the mitochondria of intestinal mucosa catalyzed by ASS1^30^. Arginine is metabolized in two ways: one is that arginine is decomposed into ornithine and urea catalyzed by arginase 1 (ARG1), the other is that arginine is metabolized into citrulline and NO catalyzed by NO synthase (NOS)^54^. In the present study, PEDV infection up-regulated ASS1 and ARG1 gene expression in the jejunal mucosa, indicating the abnormality of arginine metabolism. Besides, expression of basic amino acid transporters b^0,+^AT and y^+^LAT1 was down-regulated in PEDV-infected piglets, which may explain the reduction of basic amino acids. b^0,+^AT1 and rBAT are located in the apical membrane of epithelial cells and are responsible for transporting basic amino acids and cystine into cells from the intestinal lumen, while exchanging neutral amino acids^55,56^. y^+^LAT1 is located in the basolateral membrane of epithelial cells and is responsible for transporting basic amino acids into the blood from the intestinal cells while exchanging neutral amino acids^57^. Intriguingly, concentrations of acidic amino acids (glutamate and aspartate) were increased, while an acidic amino acid transporter SLC1A1 was down-regulated in the jejunum of PEDV-infected piglets. Wu^58^ reported that glutamate and aspartate, along with glutamine, are the major energy substrates for enterocytes. These amino acids play an important role in pig growth performance, gene expression regulation, cell signaling pathway, tight-junction integrity, hormone secretion, antioxidant capacity, and neural regulation^59–63^. The higher content of acidic amino acids in the jejunum may be due to the higher demand for these amino acids under pathological conditions. Other amino acids, including asparagine, tyrosine, and phenylalanine were increased in the PEDV group in both the jejunal mucosa and plasma, which might be related to the up-regulated expression of neutral amino acid transporters, such as B^0^AT1 and ASCT2^64^. However, glycine and cysteine (two of the three substrates for GSH synthesis), as well as proline, threonine, hydroxyproline, taurine, and alanine were decreased in the jejunal mucosa and plasma after PEDV infection, indicating disorders in protein metabolism and amino acid absorption. PepT1 is the transporter of di- and tri-peptides, locating in the brush border of the intestine^65,66^. Osmanyan *et al*.^56^ reported that the end-products of protein digestion, i.e. small peptides and amino acids, may affect the expression of PepT1. In the present study, the reduction in PepT1 expression in the PEDV group further demonstrates the abnormal metabolism of protein and amino acids in PEDV-infected piglets. Moreover, plasma urea concentration is an indicator of amino acid metabolism in the body and may be negatively correlated with piglet growth performance^67^. Therefore, PEDV infection may inhibit protein synthesis, while stimulating protein degradation and amino acid catabolism in the whole body. Taken together, PEDV infection may promote the degradation of body protein and impair intestinal amino acid absorption, transport and metabolism by altering the expression of amino acid transporters.

Moreover, lipid transport and metabolism may also be regulated by PEDV infection in piglets. PEDV could interfere with fatty acid transport by down-regulating the expression of FABP1, FABP2, and SLC27A4 in jejunal and ileal mucosae. FABP1 and FABP2 are predominantly expressed in the proximal and distal part of the small intestine, respectively^68^. Both of them play an important role in uptake, transport, and metabolism of fatty acids. SLC27A4 functions mainly as a transporter which transports long-chain fatty acids across the plasma membrane and seems to be the principal fatty acid transporter in enterocytes^69,70^. A decrease in FABP1, FABP2, and SLC27A4 expression may result in inhibition of fatty acid absorption and transport by enterocytes. However, FABP6 expression was increased by PEDV infection in the ileum of piglets. Furuhashi *et al*.^68^ showed that FABP6 could bind bile acids, the metabolites of cholesterol, and are crucial for the absorption and transport of lipids and vitamins. Therefore, PEDV can differentially regulate the expression of intestinal lipid transporters. Furthermore, PEDV may decrease the synthesis of fatty acids by down-regulating SLC27A2, ACSL3 and FASN expression in the small intestine. SLC27A2 and ACSL3 are isozymes of the long-chain fatty-acid-coenzyme A ligase family, which convert free long-chain fatty acids into fatty acyl-CoA esters, and hence play a crucial part in lipid biosynthesis and fatty acid degradation^71^. FASN catalyzes the synthesis of long-chain saturated fatty acids, which are precursors of nutritionally and physiologically significant mono-unsaturated fatty acids in animals, including pigs^67^.

Intriguingly, results of the present study indicated that lipoprotein secretion could be regulated by PEDV infection as APOA1, APOA4 and APOC2 expression were down-regulated in the jejunum and ileum of piglets. APOA1 is the main protein component of HDL in plasma and, therefore, is important for promoting cholesterol efflux from tissues to the liver for excretion^72,73^. Down-regulation of the APOA1 gene is associated with HDL deficiencies. APOC2 is the component of very low density lipoprotein. Liu *et al*.^74^ reported that APOC2 could activate LPL (an enzyme on the outer surface of endothelial cells that is responsible for the hydrolysis of plasma triglycerides), thereby playing an important role in converting triglycerides into free fatty acids for cells. APOA4 participates in the maturation of HDL and assisting APOC2 activation of LPL, and plays a vital role in the reverse transport of cholesterol. Hence, it is apparent that PEDV infection decreased the concentration of plasma HDL through the reduction of intestinal APOA1 and APOA4 expression (Fig. 4). Meanwhile, down-regulation of APOC2 may be associated with the disorder of cholesterol metabolism, and consequently result in the accumulation of cholesterol and impair enterocytes.

The IPA-derived gene networks suggested that PEDV infection could induce immune stress and intestinal dysfunction via regulating IFN-alpha, PI3K-Akt, NF-κB, and MAPK-ERK signaling pathways. Moreover, the stimulation of these pathways could induce the activation of downstream molecules such as Jnk, TCR/CD3 complex, and eventually affect nutrient transport and metabolism (mainly lipid) in the small intestine of piglets.

## Conclusion

PEDV infection caused severe diarrhea and intestinal dysfunction as indicated by increases in plasma DAO activity and i-FABP levels as well as a decrease in plasma D-xylose concentration. PEDV infection decreased expression of AQPs, amino acid transporters (y^+^LAT1, SLC1A1, b^0,+^AT, rBAT, and PepT1), altered amino acid profiles in the plasma and jejunum, affected lipid and glucose absorption and metabolism in the jejunal and ileal mucosae. These results could provide researchers with more insights in understanding the molecular mechanisms responsible for PEDV infection-induced intestinal dysfunction.

## Materials and Methods

### Animal care and diets

The experimental protocol and animal use for the present study were approved by the Animal Care and Use Committee of Wuhan Polytechnic University (2016-0323). All piglets used in the current study were purchased from a PEDV-free farm and were born at term (114 days of gestation). Sixteen crossbred healthy female pigs (Duroc × Landrace × Yorkshire, 7 days of age, with the same gender), initially weighing 2.86 ± 0.30 kg, were used in this experiment. Pigs were penned individually (2.0 × 3.0-m pens) in two rooms in a temperature-controlled nursery barn (25–27 °C). All piglets were fed a liquid milk replacer (Wuhan Anyou Feed Co., Ltd, Wuhan, China) with sufficient nutrients. The liquid diet was formulated according to the ratio of diet/water (1:4). Namely, 20 g milk replacer (powder) was dissolved in 80 mL warm water (50 ± 5 °C) to prepare the liquid feed. Each pig was fed four times per day (100 g dry matter per pig per day). This feeding strategy (more frequent provision of food at a small amount for each feeding) was adopted to prevent intestinal lesions and improve feed efficiency. Pigs had free access to drinking water. The incidence of diarrhea was observed and recorded 3 times per day throughout the whole experiment. Pig feces was classified at four levels: 0, normal; 1, pasty; 2, semiliquid; and 3, liquid^75,76^. The occurrence of diarrhea was defined as the maintenance of feces at Level 2 or Level 3 for 2 consecutive days. Then the diarrhea incidence was calculated according to the following formula: incidence of diarrhea = total number of pigs with diarrhea / (total number of pigs × experimental days) × 100%. The “total number of pigs with diarrhea” in the formula was defined as the sum of the number of pigs with diarrhea observed each day.

### Experimental design

After a 5-day adaptation period, piglets were randomly allocated to one of two groups: control and PEDV (8 pigs per group). At 8:00 pm on day 0 of the trial, piglets in the PEDV group received oral administration of PEDV (the strain from Yunnan province; kindly provided by Dr. He, College of Veterinary Medicine in Huazhong Agricultural University) at the dose of 10^4.5^ TCID50 (50% tissue culture infectious dose) per pig, whereas those in the control group were administrated with the same volume of sterile saline. The dose of PEDV (10^4.5^ TCID50) was chosen according to our previous study^9^. In order to exclude the possible effects of PEDV-induced food intake reduction on intestinal indices of piglets, the control piglets were fed the same amounts of a liquid diet as piglets in the PEDV group. On day 5 post administration of PEDV or saline, all overnight fasted piglets were orally infused 10% D-xylose (1 mL/kg BW) to determine the intestinal absorption capacity and mucosal integrity by measuring blood D-xylose concentration^77^. One hour later, jugular vein blood samples were collected, and all pigs were then sacrificed under sodium pentobarbital anesthesia (50 mg/kg BW, iv) to obtain intestinal samples^9,78–81^.

### Collection of blood samples

As mentioned earlier, all blood samples were collected from anterior vena cava of piglets at 1 h post D-xylose administration on day 5 of the trial. Blood samples were further centrifuged at 3,500 rpm for 15 min at 4 °C to obtain the supernatant fluid, i.e., plasma^9,78^, which was then stored at −20 °C until analysis.

### Collection of intestinal samples

The abdomen was opened, exposed small intestine was dissected and then placed on a chilled glass plate. The 10-cm segments were cut at the middle of the jejunum and ileum^9,78,82^. Intestinal segments (10-cm in length) were then opened longitudinally and the digesta were flushed with pre-cooled PBS^9,78,83^. After blotted dry on filter paper, intestinal mucosae were scraped into 1.5 mL sterile tubes by using a sterile glass microscope slide at 4 °C^9,49,78^, and then rapidly frozen in liquid nitrogen and stored at −80 °C until further analysis. All mucosal samples were collected within 15 min after killing.

### Blood indices

The concentrations of plasma biochemical parameters, such as blood urea nitrogen (BUN), total serum cholesterol, glucose, high density lipoprotein (HDL), low density lipoprotein (LDL), creatine kinase (CK), blood calcium, and blood chloride, were determined with commercial kits using a Hitachi 7060 Automatic Biochemical Analyzer (Hitachi, Japan)^84^. The activity of diamine oxidase (DAO) and the concentrations of D-xylose were also measured by using commercially available kits (Nanjing Jiancheng Bioengineering Institute, Nanjing, China). Additionally, intestinal fatty acid binding protein (i-FABP) in plasma was determined by using a commercial ELISA kit (Cat. No. HK 406, Hycult Biotech Inc., Wayne, PA, USA) according to the manufacturer’s instructions.

### Determination of free amino acids in plasma and jejunum by using an automatic amino acid analyzer

Amino acid profiles in plasma were determined by using an automatic amino acid analyzer (S433D, Sykam GmbH, Eresing, Germany) with minor modifications. Briefly, 0.45 mL plasma and 0.45 mL salicylsulfonic acid (5%) were adequately mixed and placed at 4 °C for 20 min. Then, the mixture was centrifuged (10,000 rpm) for 15 min, and 0.5 mL of the supernatant fluid was diluted with 0.5 mL of the lithium salt sample diluent. The final sample pH value was adjusted by adding lithium hydroxide solution and then mixed well. To exclude the possible solid substances, the mixture was further filtered through a 0.22-μm membrane into a 1.5-mL sample vial and then analyzed for free amino acids.

To determine the concentration of free amino acids in the jejunum, each frozen mucosal sample (~200 mg) was powdered under liquid nitrogen using a mortar and pestle. The powdered samples were homogenized in 1 mL salicylsulfonic acid (3%), then the mixture was centrifuged (10,000 rpm) for 15 min. Thereafter, 0.45 mL of the supernatant fluid was diluted with 0.45 mL of the lithium salt sample diluent and was then mixed. After standing at 25 °C for 10 min, the liquid mixture was also filtered through a 0.22-μm membrane into a 1.5 mL sample vial and then analyzed for free amino acids.

### Ribonucleic acid preparation and microarray analysis

Each frozen mucosal sample (~100 mg) was powdered under liquid nitrogen by using a mortar and pestle. After homogenization, total mucosal RNA was extracted following the instructions for the use of TRIzol Reagent (Invitrogen, Carlsbad, CA, USA) and was further quantified by using the NanoDrop ND-2000 UV-VIS spectrophotometer (Thermo Fisher Scientific Inc., Wilmington, DE, USA) at an OD of 260 nm. The purity of RNA was assessed by determining an OD_260_/OD_280_ ratio. Moreover, 1% denatured agarose gel electrophoresis was performed to determine RNA integrity. RNA could be used for RT-PCR analysis and microarray assay when it had a 28S/18S rRNA ratio of >1.8^85^. Only those samples that had an OD_260_/OD_280_ ratio of approximately 2.0 and showed no degradation (RNA integrity number of >7.0) were used to generate labeled targets^86^. RNA integrity number was determined using an Agilent Bioanalyzer 2100 (Agilent Technologies, Inc., Santa Clara, CA, USA). In our experiment, the ratios of OD_260_/OD_280_ were >2.0 for all samples.

Labeled fragmented single-stranded cDNAs (ss-cDNA) were synthesized by using purified total RNA (100–500 ng) as template following Affymetrix WT PLUS Labeling Assay protocols. Porcine Gene 1.1 ST Arrays (Affymetrix, Santa Clara, CA, USA) were hybridized to the biotinylated ss-cDNA targets. After 20 h of hybridization at 48 °C, arrays were washed by a fluidics station and then scanned by an imaging station in a GeneAtlas System (Affymetrix, Santa Clara, CA, USA).

After scanning, the intensity data (CEL files) of Porcine Gene 1.1 ST arrays (Affymetrix) were extracted from the image data (DAT files) by the Affymetrix Command Console Software Version 1.4, and then normalized and analyzed by the Affymetrix Transcriptome Analysis Console (TAC) Software 4.0 for gene expression profiles and DEGs. The DEGs were selected by a cutoff of fold change >1.5 at *P* ≤ 0.05.

Finally, Ingenuity Pathway Analysis (IPA) 5.5 (Ingenuity Systems Inc., Redwood City, CA, USA), a web-based pathway analysis tool, was applied to identify gene-gene interaction networks, biological functions, and canonical pathways of DEGs. The DEG datasets were uploaded into IPA software (http://www.ingenuity.com) to implement a “Core Analysis” and then the results of biofunctions, networks, and canonical pathways were generated.

### Validation of microarray results by quantitative real-time PCR

Quantitative real-time PCR (qRT-PCR) was used to verify the differentially expressed genes related to nutrient transport and to exclude any false positives in the microarray results. According to the results of network analysis, several genes were selected for verification. Total RNA was prepared as described above and reverse-transcribed using the PrimeScript RT Reagent Kit with gDNA Eraser (Takara, Dalian, China) following the instructions of the manufacturer. cDNA was synthesized and stored at −80 °C until use. Primer pairs were used for qPCR to amplify cDNA fragments. The primers were designed to span introns and intron-exon boundaries to minimize the amplification of potentially contaminating genomic DNA. The primer pairs used are shown in Supplementary File S1. The qPCR was performed using the SYBR Premix Ex TaqTM (Takara, Dalian, China) on an Applied Biosystems 7500 Fast Real-Time PCR System (Foster City, CA, USA)^86^. The total volume of PCR reaction system was 50 µL, which contained 0.2 µM of each primer, 25 µL of SYBR Premix Ex TaqTM (2x) and 4 µL of cDNA. All PCRs were performed in triplicate on a 96-well real-time PCR plate (Applied Biosystems) under the following conditions (two-step amplification): 95 °C for 30 sec, followed by 40 cycles of 95 °C for 5 sec and 60 °C for 31 sec. Moreover, the melting curve (95 °C for 15 sec, 60 °C for 1 min and 95 °C for 15 sec) with continuous fluorescence measurement was subsequently constructed for each PCR product, followed by setting at 25 °C. By analyzing the melting curves of the PCR products, the specificity of the qRT-PCR reactions was assessed^87^. Amplification efficiency was calculated based on the slope of the line: E = 10^(–1/slope)^ − 1, considering an ideal value range (0.95 ≤ E ≤ 1.05)^88^. To ensure the accuracy and sensitivity of the results obtained by qRT-PCR, sample results were normalized internally by simultaneously using the average cycle threshold (Ct) of glyceraldehyde-3-phosphate dehydrogenase (GAPDH)and ribosomal protein L4 (RPL4) as the reference genes^88–90^. The 2^−ΔΔCt^ method was used to analyze the results^90^. Each biological sample was run in triplicate.

### Statistical analysis

All the experimental data are reported as means with SD. Differences between groups are analyzed by the independent two-sample *t*-test using the SPSS 17.0 software (SPSS Inc. Chicago, IL, USA). The incidence of diarrhea was analyzed by *χ*^2^ analysis. Following the 2^−ΔΔCt^ method, the mean value of jejunal and ileal gene expression of piglets in the control group was set to 1.00. Additionally, the Levene’s test was used to test the normality and constant variance for all data^91^. Possibility value ≤ 0.05 was taken to be statistically significant^92^.

### Ethics statement

The animal experiment was carried out in accordance with the Chinese Guidelines for Animal Welfare and Experimental Protocol, and was approved by the Animal Care and Use Committee of Wuhan Polytechnic University.

## Supplementary Information

Supplementary Information

Supplementary Information 2

Supplementary Information 3

Supplementary Information 4

## Data Availability

We declare that we support data availability, which allows unlimited access to our published materials, data and associated protocols promptly available to readers.
